# King Edward VII's Hospital, Cardiff

**Published:** 1913-02-22

**Authors:** 


### KING EDWARD VII'S HOSPITAL, CARDIFF.

The February meeting of the Board of Management of this hospital had many features of interest. Commenting on the number of patients waiting, Colonel Bruce Vaughan appealed to everyone, and especially working men and wealthy docksmen, to help by generous contributions to enable the Board of Management to open the remaining fifty beds in the new wing. Over 800 poor people, it was stated, including 340 women and 230 children, waited in daily anxiety for the long-expected call to the hospital.

A proposal to institute a Massage Department met with unanimous approval, and it was decided to appoint a resident masseuse sister, who should be both a fully trained nurse and a qualified and trained masseuse. Owing to the development of the hospital, it was decided, upon the recommendation of the Medical Board, to appoint a sixth resident medical officer. Colonel Bruce Vaughan said he would approve the appointment if it ensured greater efficiency, but he considered the time was ripe for a systematised arrangement of the work and leisure of the residents, after the methods of the London hospitals.

The report of a Special Committee appointed to consider the position of the hospital, having regard to the provisions of the National Health Insurance Act, was unanimously adopted. The report was to the effect that, while deprecating any alteration in the existing methods of receiving patients, which might possibly lead to hardship, owing to lack of experience of the actual effects of the Act and the difficulties incidental to its initiation, the interests of the hospital and of the patients would best be served by a strict adherence to the existing regulation—namely, "Minor accidents and urgent cases will be attended to, in the first instance, without inquiry, but transferred to their own medical attendant subsequently if not eligible for treatment under the rules of the hospital"—and further by providing on out-patient tickets a form of medical certificate similar to that now in use on in-patient tickets. This certificate will only be required in the case of patients already provided with medical practitioners. It was also decided to ask subscribers to use their efforts to ensure that patients suffering from minor illnesses should be seen by their own medical practitioners, and that the decision of the Board of Management on the whole matter be made known to the doctors on the panels.

